# Distinct host immune responses in recurrent vulvovaginal candidiasis and vulvovaginal candidiasis

**DOI:** 10.3389/fimmu.2022.959740

**Published:** 2022-07-28

**Authors:** Gai Ge, Zhiya Yang, Dongmei Li, Ning Zhang, Biao Chen, Dongmei Shi

**Affiliations:** ^1^ Institute of Dermatology, Chinese Academy of Medical Sciences and Peking Union Medical College, Nanjing, China; ^2^ Laboratory of Medical Mycology, Jining No.1 People’s Hospital, Jining, China; ^3^ Department of Microbiology & Immunology, Georgetown University Medical Center, Washington DC, United States; ^4^ Department of Dermatology, Jining No.1 People’s Hospital, Jining, China

**Keywords:** recurrent vulvovaginal candidiasis (RVVC), vulvovaginal candidiasis (VVC), host-immune responses, T helper cells (Th), anti-fungal susceptibility

## Abstract

Recurrent vulvovaginal candidiasis (RVVC) and vulvovaginal candidiasis (RVVC) are one of the most common gynecological infections, primarily caused by *Candida* species. Although risk factors of RVVC and VVC have been identified in many studies, antifungal immunological mechanisms are still not fully understood. We performed a 1-year prospective study in a local hospital to monitor 98 patients clinically diagnosed with gynecological *Candida* infection. The results showed that 20.41% (20/98) are with RVVC, and 79.59% (78/98) patients have VVC. *C. albicans* accounts for 90% and 96.1% of all strains isolated collected from RVVC and VVC patients, respectively. Antifungal susceptibility testing showed no significant difference in *Candida* species between RVVC and VVC patients. However, the serum levels of IFN-γ, TNF-α, and IL-17F in the RVVC group were significantly lower than those of the VVC group, while IL-4, IL-6, and IL-10 were higher in the RVVC patients than VVC patients. IL-17A and IL-2 levels were comparable between the two groups. Taken together, our results suggest that the host-immune responses, especially Th1/2 immunity, may play important roles in prognosis of RVVC and VVC.

## Introduction

Vulvovaginal candidiasis (VVC) is one of the most prevalent manifestations of superficial fungal infections, commonly presented by vaginal and vulvar pruritus, burning and irritation, dyspareunia, and abnormal vaginal discharges (1–3). VVC affects up to 75% of women of childbearing age at least once during their lifetime (4, 5). Recurrent vulvovaginal candidiasis (RVVC) is found in up to 9% of the general women population (6), defined as the patient experiencing four or more episodes of infection per annum. Compared with VVC, RVVC may more severely affect the patients’ life quality and impose a more significant economic burden. RVVC can further cause patients to lose their confidence and self-esteem, which will affect not only their abilities for daily physical activities but also sexual life and intimate relationships (4, 5, 7). Currently, approximately 138 million women suffer from RVVC annually, and this number is expected to increase to 158 million by 2030 (4).

**Figure 1:**
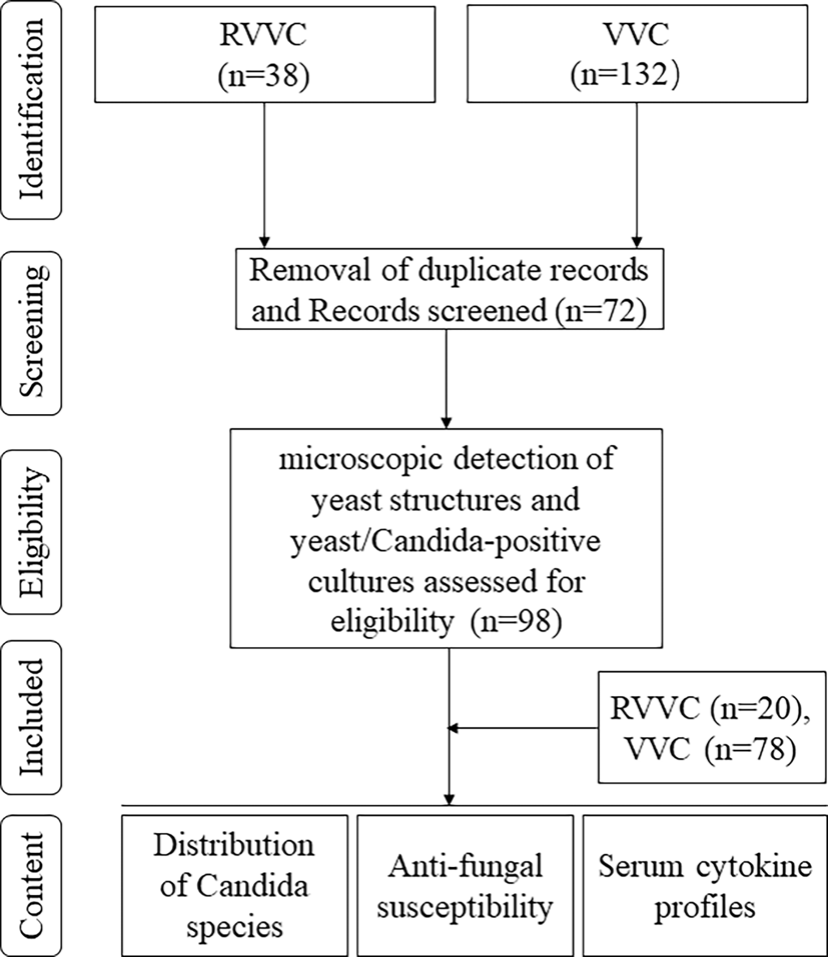
Flowchart of the study.

The pathogenesis of RVVC and VVC has been related to dysbiosis of the microbiome in the vagina, host-immune response, pathogen virulence, antifungal drug resistance, and other factors (8). However, host-immune responses may play important roles in prognosis of RVVC and VVC. Rosati et al. even considered that RVVC could be an immunodeficient or autoinflammatory disease (7). CD4+ T cells and their cytokines play a central role in antifungal immunity, in which Th1 and Th17 cells are the principal effector cells responsible for protective immunity while Th2 responses are thought to be associated with deleterious effects (9). In this study, we investigated and compared the differences in *Candida* distribution, antifungal drug resistance, and immune responses between RVVC and VVC (**Figure 1**).

## Materials and methods

### Including and excluding criterion of the patients

The protocol of this study was approved by the Research Ethics Committee of the Jining No. 1 People’s Hospital. One hundred seventy patients with signs and symptoms of vaginal infection were recruited at the beginning. Informed consent was obtained from all patients involved. The patients filled in a questionnaire about general information, episodes of infection, past and current underlying diseases, and treatment history. Specimens collected from these patients were then proceeded for fluorescent calcofluor white (CFW) staining and culture on Sabouraud Dextrose Agar (SDA) for 48 h at 35°C. After mycological confirmation, 98 patients were enrolled in this study (**Figure 1**). They were divided into RVVC and VVC groups according to the diagnostic standards (6). Inclusion criteria were the following: microscopic detection of yeast structures or yeast cells and *Candida*-positive cultures for VVC (4, 10); the patient experienced four or more episodes of VVC per annum for RVVC (6); and menstruation ending at least 3 days by the time of diagnosis. The exclusion criteria were the following: pregnant women and women with diabetes, immunosuppression, or having antifungal treatment within 1 month or vaginal irrigation or vaginal insert within 7 days prior to the study.

### The isolation and molecular identification of yeast strain isolated from patients with RVVC or VVC

The purified yeast colonies were identified by the morphologic method. The *Candida* species were then identified by sequencing the internal transcribed spacer region of ribosomal DNA (ITS). Genomic DNA was isolated from the strain by mechanical homogenization in lysis buffer (E.Z.N.A.^®^ Tissue DNA Kit D3396, OMEGA, USA). The ITS region was amplified in each isolate using the following primes: ITS1 (5′-TCCGTAGGTGAACCTGCGG-3′) and ITS4 (5′-TCCTCCGCTTATTGATATGC-3′). The amplification program was schematically represented in the following way: 30 s 98°C; 35× [10 s 98°C, 30 s 57°C, 5 min 72°C]; 5 min 72°C; temporary storage at 4°C. The obtained sequences were compared to the NCBI nucleotide database (https://blast.ncbi.nlm.nih.gov/Blast.cgi) and the Westerdijk Fungal Biodiversity Institute (CBS) database (http://www.cbs.knaw.nl) to verify the species-level identity of each isolate.

### Antifungal susceptibility testing

The purified colonies from the patients were tested for *in vitro* antifungal susceptibility according to the CLSI reference guideline M27-Ed4 and M27-S4 (11). The tested antifungals included eight antifungal agents: voriconazole (VRC; Sigma-Aldrich, USA), posaconazole (POS; Sigma-Aldrich, USA), amphotericin B (AmB; Sigma-Aldrich, USA), terbinafine (TER; Sigma-Aldrich, USA), ketoconazole (KET; Sigma-Aldrich, USA), fluconazole (FLC; Sigma-Aldrich, USA), itraconazole (ITC; Sigma-Aldrich, USA), and micafungin (MFG; J&K Scientific, China). For all experiments, *C. parapsilosis* ATCC 22019 was used as a control strain (11). Each isolate was subcultured onto SDA at 35°C for 24–72 h to verify its viability before minimum inhibitory concentration (MIC) measurement. Colonies were suspended in sterile saline, and the final inoculum concentration of the suspension was adjusted to 5 × 10^3^ CFU ml^-1^ in RPMI 1640 medium. The 96-well plates were incubated for 24 or 48 h at 35°C, and the MIC levels were determined visually. The drug concentration ranges, MIC reading points, and interpretive breakpoints used for eight antifungal agents are listed in **Table 1**. Although the interpretive criteria for the susceptibility to AmB remain controversial, we here classified MIC ≤1 μg/ml as susceptible and MIC ≥ 2 μg/ml as resistant, referring to previous studies (12). There are no interpretive breakpoints for TER.

**Table 1:** Drug concentration range, time of MIC reading, and interpretive breakpoints for eight antifungal agents.

Drug	Ranges (μg/mL)	MIC time	*C. albicans* MIC (μg/mL)			*C. glabrata* MIC (μg/mL)		
			*S*	*SDD*	*R*	*S*	*SDD*	*R*
VRC	0.0313-16	48h	≤0.125	0.25-0.5	≥1	–	–	–
POS	0.0313-16	48h	≤1	-^a^	–	–	–	–
KET	0.0313-16	24h	≤0.125	0.25-0.5	≥1^[11]^	–	–	–
AMB	0.0313-16	24h	≤1	–	≥2	–	–	–
TER	0.0313-16	24h	–	–	–	–	–	–
FLC	0.125-64	24h	≤2	4	≥8	–	≤32	≥64
ITC	0.0313-16	48h	≤0.125	0.25-0.5	≥1	–	–	–
MFG	0.016-8	24h	≤0.25	0.5	≥1	≤0.06	0.12	≥0.25

a Not applicable.

S, susceptible; SDD, susceptible-dose dependent; R, resistant.

### The analysis of cytokines in the serum of the patients

The patients’ blood was collected for detecting the cytokines in the serum prior to treatment. The levels of IFN-γ, IL-2, TNF-α, IL-6, IL-17A, IL-17F, IL-4, and IL-10 in each serum specimen of patients were measured, respectively. Serum cytokine profiles were measured using a bead-based multiplex assay (LEGENDplex™; BioLegend, San Diego, CA, USA), according to the manufacturer’s protocols.

### Statistical analyses

Analysis of data was performed using SPSS software (version 22), Chi-square test, Mann–Whitney test, and two-tailed Student’s t-test. GraphPad Prism 8 (GraphPad, San Diego, CA, USA) was used for drafting. All results were expressed as mean ± standard deviation (SD) (pg/mL or ng/mL).

## Results

### Prevalence of RVVC and VVC in women at ages 25–34 years

**Figure 2:**
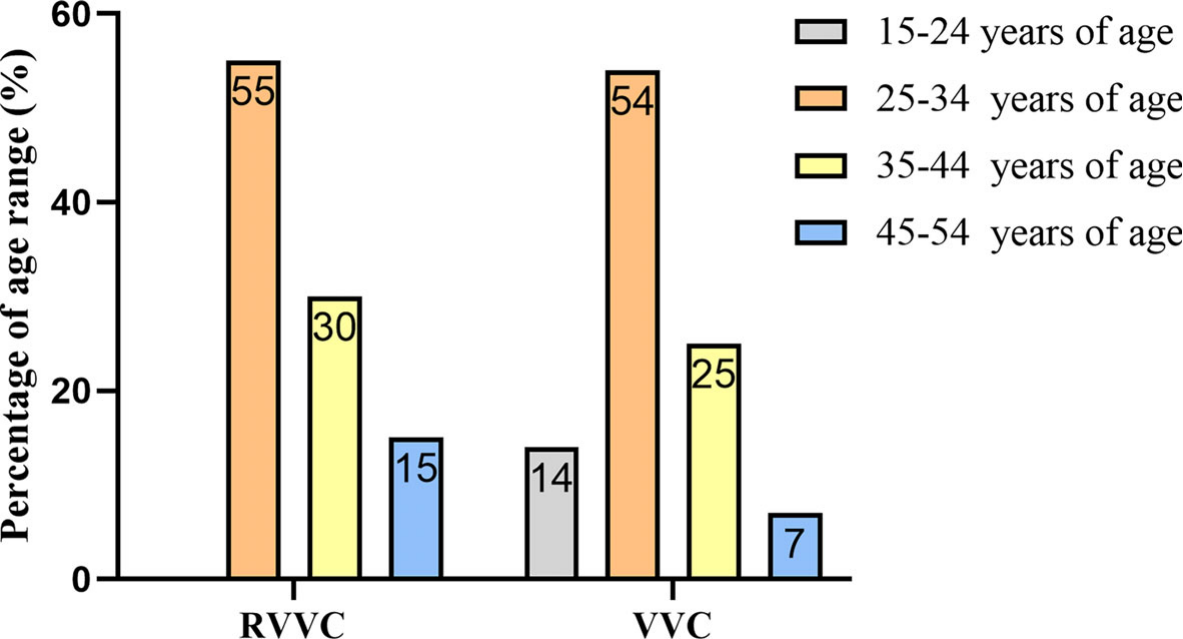
Age distribution of women with recurrent vulvovaginal candidiasis (RVVC) and vulvovaginal candidiasis (VVC).

During the study period, 170 patients with signs and symptoms of vaginal infection were evaluated. Of them, 98 (57.64%) were diagnosed with RVVC and VVC according to diagnostic standards. The average ages in the RVVC group and VVC group were 35.1 and 31.3 years, respectively. The age range in the RVVC group was as follows: 15–24 years, (n = 0), 25–34 years (n = 11, 55.0%), 35–44 years (n = 6, 30.0%), and 45–54 years (n = 3, 15.0%). In the VVC group, 15–24 years accounted for 14.0% (n = 11), 25–34 years for 54.0% (n = 41), 35–44 years for 25.0% (n = 19), and perimenopausal age 45–54 years for 7.0% (n = 5). Although the onset ages and mean age of the VVC group appear younger than those of the RVVC group, more than 50% of participants in both groups fall in reproductive ages (*p >* 0.05) (**Figure 2**).

### Antibiotic treatment induces RVVC and VVC

External risk factors for RVVC and VVC infection were evaluated by analyzing medical treatment history, clinical symptoms, and any other concurrent infections. We found that antibiotic therapies with azithromycin, clindamycin, or cephalosporin—or antifungal metronidazole therapies—correlated well with candidal vaginitis (*p* < 0.05). However, symptomatic factors including discomfort after sexual activity, redundant prepuce, genital itching, chlamydia, and mycoplasma infections had no apparent impact on RVVC or VVC (*p >* 0.05). Other medical conditions such as gastritis, allergic rhinitis, and skin disorders such as tinea pedis also had no effect on RVVC or VVC (*p >* 0.05). We also found that family history (i.e., mother or daughter with a history of RVVC or VVC) accounted for 5% and 5.3%, respectively (*p* > 0.05), of our sample. Previous use of antifungal agents such as nysfungin and clotrimazole, for previous episode(s) of RVVC or VVC, had no apparent impacts on recurrence (*p* > 0.05) (**Table 2**).

**Table 2:** Risk factors of the RVVC group and VVC group.

Risk factors /Underlying diseases		RVVC	VVC	*P*
	Unknown	3 (15%)	9 (11.8%)	0.969
	Antibiotics	4 (20%)	2 (2.6%)	0.017
	discomfort after sexual practices	1 (5%)	3 (3.9%)	0.295
	genitals itching Candidal balanoposthitis	4 (20%)	8 (10.5%)	
	redundant prepuce	1 (5%)	4 (5.3%)	
	chlamydia and mycoplasma infections	2 (10%)	3 (3.9%)	0.585
	family history	1 (5%)	4 (5.3%)	1
	tinea pedis	–	4 (5.3%)	–
	other internal medicine	5 (25%)	13 (17.1%)	0.593
Treatment		RVVC	VVC	
	Nifuratel	10 (50%)	15 (19.7%)	0.005
	Nysfungin	7 (35%)	16 (21.1%)	0.173
	Clotrimazole	5 (25%)	12 (15.8%)	0.495
	Fluconazole	2 (10%)	4 (5.3%)	0.773
	Miconazole	2 (10%)	2 (2.6%)	0.386
	itraconazole	–	1 (1.3%)	–
	griseofulvin	1 (5%)	–	–
	Others	5 (25%)	9 (11.8%)	0.239

### Distribution of *Candida* species is not a reason for the clinical difference between RVVC and VVC

**Figure 3:**
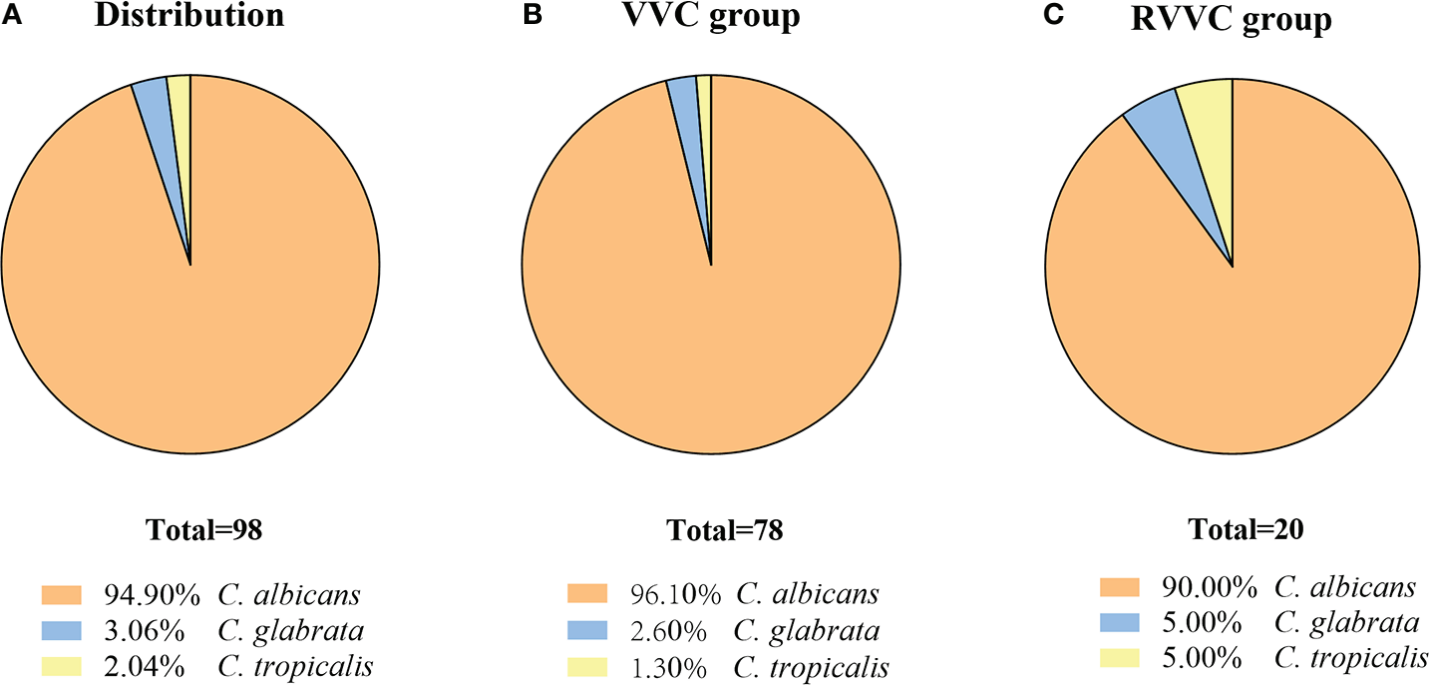
The isolates from vaginal secretion were identified by ITS sequencing. The Candida spp. distributed in both Vulvovaginal candidiasis (VVC) patients and Recurrent vulvovaginal candidiasis (RVVC) patients **(A)**, in VVC patients **(B)** and in RVVC patients **(C)**.

Among the vaginal specimens, 98 out of 170 Candida isolates were identified as *C. albicans*, *C. glabrata*, and *C. tropicalis*. Based on the sequencing results of 98 isolates, 93 isolates were *C. albicans* (94.9%), three strains were *C. glabrata* (3.1%), and two isolates were *C. tropicalis* (2%) (**Figure 3A**). In 78 strains isolated from the VVC group, 96.10% (75/78) were *C. albicans*, and the rest of the strains were *C. glabrata* (n = 2) and *C. tropicalis* (n = 1) (**Figure 3B**). Among a total of 20 isolates from the RVVC group, *C. albicans* accounted for 90.00% (n = 18), *C. glabrata* for 5.00% (n = 1), and *C. tropicalis* for 5.00% (n = 1) (**Figure 3C**). The proportion (10%) of non-albicans Candida (NAC) species was higher in the RVVC group than that in the VVC group (3.9%), but there was no statistical difference (*p* > 0.05).

### Antifungal susceptibility profiles of the RVVC group were consistent to the VVC group

MIC distribution, MIC 50/MIC 90, geometric mean values (GM), susceptibility rate (S), susceptible dose-dependent rate (SDD), and resistant rate (R) of 93 C*. albicans* and five NAC isolates were measured for eight antifungal agents; the results are summarized in **Tables 3**, **4**.

**Table 3:** The *in vitro* antifungal susceptibility of *C. albicans* isolated from patients with RVVC or VVC to eight antifungal drugs.

Drugs		MIC (μg/ml)					
		Ranges	MIC_50/90_	GM	S%	SDD%	R%
VRC	RVVC	<0.0313-1	0.0313/0.25	0.095	89	5.5	5.5
	VVC	<0.0313-0.5	0.0313/0.125	0.075	90.7	9.3	0
POS	RVVC	<0.0313-0.5	0.0313/0.25	0.063	100	0	0
	VVC	<0.0313-2	0.0313/0.25	0.055	97.3	–	–
AMB	RVVC	1 to 8	2 to 4	2.619	11.1	0	88.9
	VVC	1 to 8	4 to 8	3.924	1.3	0	98.7
KET	RVVC	<0.0313-1	0.0625/0.5	0.105	66.7	27.8	5.5
	VVC	<0.0313-1	0.0625/0.5	0.116	73.4	21.3	5.3
TER	RVVC	4->16	>16/>16	–	–	–	–
	VVC	1->16	>16/>16	–	–	–	–
FLC	RVVC	<0.125-8	0.5/4	0.52	83.3	11.1	5.6
	VVC	<0.125-16	1 to 2	0.583	92	2.7	5.3
ITC	RVVC	0.0313-0.5	0.0625/0.5	0.107	61.1	38.9	0
	VVC	<0.0313-8	0.0625/2	0.125	64	21.3	14.7
MFG	RVVC	0.016-0.125	0.016/0.0313	0.02	100	0	0
	VVC	0.016-0.0625	0.016/0.0313	0.019	100	0	0

S, susceptible; SDD, susceptible-dose dependent; R, resistant.

**Table 4:** The *in vitro* antifungal susceptibility of NAC isolated from patients with RVVC or VVC to eight antifungal drugs.

Drugs	MIC (μg/mL)				
	*C. glabrata* (n=3)			*C. tropicalis* (n=2)	
VRC	0.125	0.0313	0.0625	0.0625	0.0313
POS	1	0.0313	0.0313	0.0313	<0.0313
AMB	4	4	4	2	8
KET	0.125	0.0625	0.25	0.25	0.0313
TER	>16	>16	>16	>16	>16
FLC	8	1	1	0.25	0.125
ITC	2	0.25	0.0313	0.0313	0.0313
MFG	0.0625	0.016	0.0313	0.0625	0.016

Based on these results, the susceptibility rates of *Candida* isolates in the RVVC or VVC group to VRC, POS, AMB, KET, FLC, ITC, and MFG were 89%, 100%, 11.1%, 66.7%, 83.3%, 61.1%, 100%, and 90.7%, 97.3%, 1.3%, 73.4%, 92%, 64%, 100%, respectively. Moreover, the resistance rates in the RVVC or VVC group to VRC, POS, AMB, KET, FLC, ITC, and MFG were 5.5%, 0%, 88.9%, 5.5%, 5.6%, 0%, 0%, and 0%, 0%, 98.7%, 5.3%, 5.3%, 14.7%, 0%, respectively. Regarding GM values and sensitivities, *C. albicans* to POS, KET, FLC, and ITC were consistent between the VVC and RVVC groups. For the VRC agent, GM values were slightly higher in isolates of the RVVC group than those of the VVC group, but the sensitivity was more reduced in the RVVC isolates than the VVC isolates. However, these differences in VRC response between VVC and RVVC isolates had no statistical significance (*p* > 0.05). In the RVVC group, the GM values of *C. albicans* to AmB were lower than those strains from VVC, but the sensitivity was higher with statistical significance (*p* < 0.05). Higher GM values of *C. albicans* to MFG were also shown in the RVVC group with 100% sensitivity in isolates from both groups. In summary, *C. albicans* isolates were sensitive to MFG, and their sensitivities to VRC, POS, and FLC were higher than those to KET and ITC, but they were less susceptible to AmB and resistant to TER *in vitro*.

In addition, *C. glabrata* was 100% susceptible to FLC and MFG. However, the MIC_50/90_ of *C. glabrata* isolates to other drugs except AmB and TER were higher than those of *C. albicans.* Since a small number of *C. tropicalis* strains were collected in this study, and reading points of *in vitro* antifungal susceptibility for most NAC strains remain incomplete, the MIC values of two strains of *C. tropicalis* to eight antifungal drugs were not determined in this study.

### The immune responses, especially Th1 immunity, may contribute to the clinical difference between RVVC and VVC

**Figure 4:**
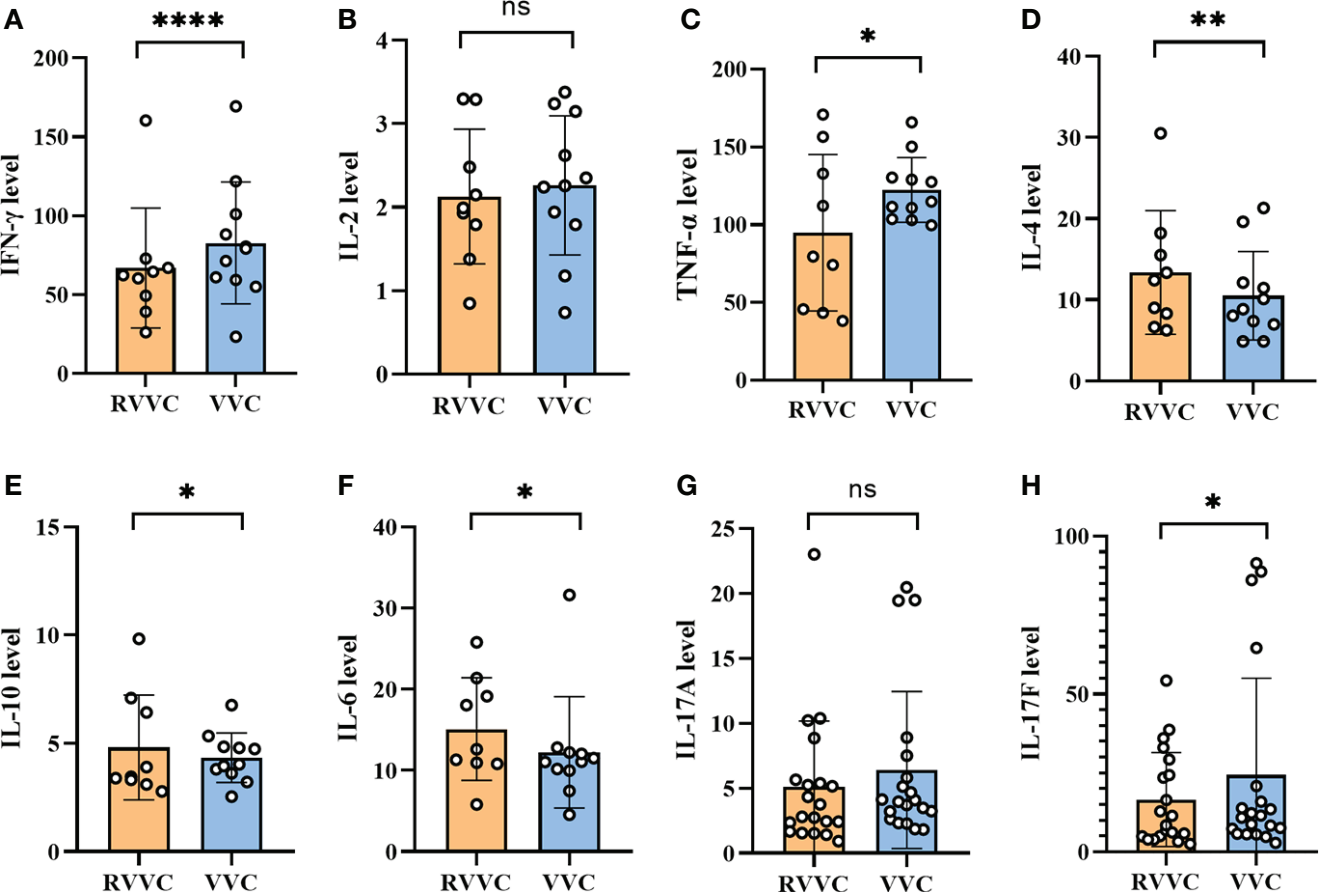
The serum was collected from the VVC and RVVC patients before the initiation of treatment to analyze the cytokines in the serum by LEGENDplex™. **(A-C)** represent the serum level of IFN-γ, IL-2 and TNF-α (Th1 associated cytokines) of the patients with Recurrent vulvovaginal candidiasis (RVVC) and Vulvovaginal candidiasis (VVC), respectively. **(D, E)** represent the serum level of IL-4 and IL-10 (Th2 associated cytokines) of the patients with RVVC and VVC, respectively. **(F-H)** represent the serum level of IL-6, IL-17A and IL-17F (Th17 associated cytokines) of the patients with RVVC and VVC, respectively. Data are representative of three independent experiments. P value < 0.05 is regarded as a significant enrichment. Error bars represent SD, *p < 0.05, **p < 0.01, ***p < 0.001. ns, No significance.

The serum levels of IFN-γ, TNF-α, IL-4, IL-10, IL-6, IL-17A, and IL-17F in the RVVC or VVC group were 66.98 pg/ml, 2.129 pg/ml, 94.89 pg/ml, 12.4 pg/ml, 3.46 pg/ml, 12.6 pg/ml, 3.28 ng/m, and 9.95 ng/ml, 82.81 pg/ml, 2.263 pg/ml, 122.5 pg/ml, 8.86 pg/ml, 4.03 pg/ml, 11.07 pg/ml, 4.03 ng/m, and 11.23 ng/ml, respectively. Summarily, the serum levels of IFN-γ, TNF-α, and IL-17F in the RVVC group were lower but those of IL-4, IL-6, and IL-10 were higher than those in the VVC group with statistical significance (*p* < 0.05). However, the levels of IL-17A and IL-2 exhibited no statistical significance (*p* > 0.05) between the VVC and RVVC groups (**Figure 4**).

## Discussion

We performed a 1-year prospective study in a local hospital to compare clinically diagnosed RVVC and VVC patients for *Candida* distribution, antifungal drug resistance, and immune responses. Age seems to have been an important factor overall in the occurrence of vulvovaginal candidiasis (4) in this study. We find that the prevalence of VVC and RVVC in women aged 25–34 is similar to that reported by Annabel Lines et al. (6). VVC and RVVC are considered multifactorial disorders, in which other vaginal infections, broad-spectrum antibiotic therapies, colds, diabetes, oral contraceptives, and intrauterine devices all favor disease onset (13). External factors include redundant prepuce, discomfort after sexual activity, and reinfections by untreated partners. In this study, we find that up to 30% of RVVC patients had one or more of the above problems. In a very recent self-reported study, Benedict et al. reported that 5.2% (98/1869) of women respondents in the USA had VVC, and of those, 4.7% (5/98) were RVVC (14). This compares strikingly with 53% (43/81) of VVC patients in Iran who experienced RVVC in a study by Arastehfar et al. (8), while we found 11.76% (20 cases) of RVVC in 170 patients with signs and symptoms of vaginal infection. There are many reasons that may explain such a wide variation in RVVC prevalence beyond race and national origin. Age, internal and external factors noted above, and species distribution may all work together to induce RVVC and VVC.

The source of recurrent episodes of RVVC and VVC is unclear. Given that *C. albicans* is considered an opportunistic pathogen with a prolonged colonization state in the host before active infection, such a colonization phase could play a vital role in the pathogenesis of idiopathic RVVC (7). Tian et al. found that multiple isolates from 26 (59.1%) RVVC patients shared the same genotype (15). Pappas et al. (16) suggested a few mechanisms, in particular reinfections of exogenous origin from the gastrointestinal tract or infected partners or incomplete eradication of microorganisms by inadequate or improperly terminated treatment. Since *C. albicans* has been found to remain in the vagina between clinical episodes (17), Cassone et al. (18) proposed a possible mechanism whereby the symptoms are due to an allergic response to a component of yeast-state *C. albicans*.

The NAC (non-*albicans Candida*) species have recently sparked scientific and epidemiological interest as their prevalence keeps increasing globally (19). These NAC species should be a matter of concern during the treatment of RVVC patients due to their propensity for drug resistance (8). Although NAC species have been more commonly found in RVVC (20, 21), *C. albicans* and *C. glabrata* account for more than 90% of yeast isolates in our study, which agrees with other reports (8, 22, 23). The portions of NAC species in VVC cases have been reported as 41.4% (n = 87) in Ethiopia (24) and 10%–30% in one other study (25). Furthermore, our result was comparable with the previous observation, in which the presence of a burning sensation is reported in women with *C. glabrata* and itching with *C. albicans* infections (19).

The treatment option for sporadic VVC and RVVC relies on the administration of antifungal agents that often requires long-term regimens in order to keep vaginal fungal burden to a controllable level (26). For a *C. albicans* infection or empirical treatment, fluconazole is often scheduled in two phases: the induction phase (oral fluconazole 150 mg, once every 3 days for three doses) and the maintenance phase (oral fluconazole 150 mg once a week for 6 months). Up to 90% clinical remission can be achieved in patients treated with this regimen (6, 27). If fluconazole is contraindicated or not tolerated, a topical imidazole treatment for 7–14 days can be an alternative. According to response, clotrimazole 500 mg intravaginally once a week or oral itraconazole 50–100 mg daily can supplement the maintenance treatment. Oral antifungal therapy should be avoided in women who are pregnant or breastfeeding. For NAC infections, 6 months of nystatin 100,000 units intravaginally for 14 consecutive nights per month is recommended. Although most NAC are *C. glabrata* which is susceptible to azoles, recent evidence suggests that topical nystatin is equally effective against *C. glabrata* (28). Boric acid is another alternative, but it is rarely used in primary care because of its lack of availability through community pharmacies (29). In the case of RVVC treatment, systemic FLC and triazoles (ITC) are typically coupled with a local application of imidazole (5, 30). According to the Iranian data noted above, the multifactorial nature of RVVC includes host- and drug-related factors such as FLC-resistant and FLC-tolerant isolates (8). Triazoles, polyenes, nucleosides, acrylamides, and echinocandins are still effective drugs in the treatment of VVC. Studies have also shown that AmB, FLC, and nystatin are highly effective to treat *C. albicans*-infected VVC with 70%–98%, 95%, and 95% cure rates, respectively. The sensitivity data of *C. albicans* to VRC, POS, and KET in this study vary from those of other reports in China (12, 31–33) but agree with one foreign study (34). MFG is the most active drug against *C. albicans* isolated from RVVC and VVC in our results, but our isolates are most resistant to TER, followed by AmB and ITC. In this study, in addition to initially infected women, each patient had a history of antifungal therapy, and more than half of the patients had received more than one type of antifungal treatment, especially the RVVC patients.

Recently, it has been confirmed that genetic defects of Th17 or its related cytokines can promote the occurrence and development of cutaneous mucosal *C. albicans* infections such as chronic mucocutaneous candidiasis (CMC) (35). CMC often appears in patients with profound primary T-cell immunodeficiency. However, for an immunocompetent host, data regarding the cell-mediated immune response in modulating the host response to RVVC are limited and not confirmed. RVVC could also be an immunodeficient condition or autoinflammatory disease (7). CD4^+^ T cells and their cytokines are known to play a central role in antifungal immunity, in which Th1 and Th17 cells are the principal effector cells responsible for protective immunity against fungi while Th2 responses are thought to be associated with various deleterious effects (9). However, in an experimental estrogen-dependent murine model, there is no systemic T-cell infiltration into the vaginal mucosa and normal levels of Th1/Th2 cytokines are found in both RVVC patients and controls (36), suggesting that RVVC and VVC are not immunodeficiency-associated conditions (37). Our data support the hypothesis that T-cell immunity could provide protection from the recurrence of fungal vaginitis, which is demonstrated by the difference in cytokine responses between RVVC and VVC. We found that the immune responses of the VVC patients favored the activation of the IFN-γ-produced Th1 and IL-17-secreted Th17 cells. Both cytokines were less abundantly produced in RVVC, but IL-4 is highly produced in RVVC patients, suggesting a likely Th2 response in RVVC. Nevertheless, the cytokine responses may suggest that the recurrent preference in RVVC could be a consequence of the lower Th1 and Th17 responses.

## Conclusions

In conclusion, RVVC and VVC are prevalent in women aged 25–34 years, caused mainly by *C. albicans* and less by *C. glabrata*. There is no genotypical strain correlated with the clinical difference between RVVC and VVC. From the perspective of host immunology, the Th1/Th2 balance may play an important role in RVVC.

## Data availability statement

The data presented in the study are deposited in the GenBank repository, accession numbers ON907664 to ON907761.

## Ethics statement

The studies involving human participants were reviewed and approved by the Research Ethics Committee of the Jining No.1 People’s Hospital. The patients/participants provided their written informed consent to participate in this study.

## Author contributions

DS and DL conceived and designed the research. GG and NZ performed the experiments. GG wrote the manuscript. ZY and BC evaluated the experimental data. All authors reviewed and discussed the manuscript.

## Funding

This work was supported in part by grants from the National Natural Science Foundation of China (NM.81773337) and the Development Plan of Jining (NM 2021YXNS121), Shandong, China.

## Acknowledgments

The authors acknowledge the contributions of all the scientists in this area and apologize for failing to cite any work due to constraints of space.

## Conflict of interest

The authors declare that the research was conducted in the absence of any commercial or financial relationships that could be construed as a potential conflict of interest.

## Publisher’s note

All claims expressed in this article are solely those of the authors and do not necessarily represent those of their affiliated organizations, or those of the publisher, the editors and the reviewers. Any product that may be evaluated in this article, or claim that may be made by its manufacturer, is not guaranteed or endorsed by the publisher.
